# In vitro tyrosinase inhibitory, DNA interaction studies, and LC–HRMS analysis of *Ficus carica* leaves

**DOI:** 10.55730/1300-0527.3552

**Published:** 2023-02-28

**Authors:** Gülin RENDA, Burak BARUT, Rümeysa CEREN, Enes AYDIN

**Affiliations:** 1 Department of Pharmacognosy, Faculty of Pharmacy, Karadeniz Technical University, Trabzon, Turkey; 2 Department of Biochemistry, Faculty of Pharmacy, Karadeniz Technical University, Trabzon, Turkey

**Keywords:** *F. carica*, LC-HRMS, plasmid pBR322 DNA, rutin, tyrosinase inhibitory

## Abstract

Turkey is the world’s leading producer of figs, a typical Mediterranean fruit. The fig, *Ficus carica* L. (Moraceae), has been widely cultivated since ancient times due to the nutritional value of its fruits. It was aimed to investigate the phytochemical characterization and biological properties of *F. carica* leaf extracts in order to determine their potential for use in the treatment of various diseases. *F. carica* leaves were extracted in 70% methanol at 40 °C under reflux. To obtain extracts of different polarities, the crude extract was fractionated with *n*-hexane, dichloromethane, and *n*-butanol. Phenolic content was determined using liquid chromatography–high resolution mass spectrometry (LC–HRMS). 2,2-diphenyl-1-picrylhydrazyl (DPPH) radical scavenging and antityrosinase activities of all extracts were investigated using spectrophotometric methods. Furthermore, the DNA-damage protective properties of extracts were investigated using electrophoretic methods. The *n*-butanol extract was found to have the highest total phenolic content, with 72.58 ± 4.52 mg GAE/g dry weight. According to LC–HRMS analysis, rutin (40.13 g/kg) was the most abundant compound in the *n*-butanol extract. The *n*-butanol extract, which was found to have the highest tyrosinase inhibitory effects among the extracts, demonstrated radical scavenging activity of 37.01 ± 1.15% and 82.57 ± 0.88% at 80 and 200 μg/mL, respectively. The *n*-butanol extract had the highest protective effects against Fenton’s reagent, UV radiation, and singlet oxygen. Given these findings, it is possible to argue that *F. carica* leaves can be evaluated for developing products that could be used to treat various diseases.

## 1. Introduction

*Ficus carica* L. belongs to the family Moraceae and grows in Turkey, Morocco, Egypt, Spain, Greece, California, Italy, and Brazil [1,2]. *F. carica* leaves, fruits, and roots are used in the natural medicinal system for the treatment of gastrointestinal (colic, indigestion, anorexia, and diarrhea), respiratory (sore throat, cough, and bronchial problems), inflammatory, and cardiovascular diseases [3]. Phytochemical studies on *F. carica* have focused on phytosterols, anthocyanins, amino acids, organic acids, fatty acids, phenolic compounds, hydrocarbons, aliphatic alcohols, and volatile compounds isolated from various parts of the plant. These phytochemicals were found primarily in latex, leaves, fruit, and roots [4]. Several phytochemical components of *F. carica* are used in the production of sunscreen and coloring agents [5]. Using the ferric reducing antioxidant method, Çalışkan and Polat reported the antioxidant properties of *F. carica* [6]. Solomon et al. investigated the total flavonoid, antioxidant capacity, and profile properties of anthocyanins in the fruits of *F. carica* [7]. Acidified methanolic extract of *F. carica* peels’ tyrosinase, alpha-glucosidase, urease, and cholinesterases enzyme inhibitory effects were studied [8]. As a result of these studies, it has been reported that the fruits have high antioxidant properties due to their high content of polyphenols, flavonoids, and anthocyanins. Rubnov et al. isolated a mixture of 6-*O*-acyl-*β*-D-glucosyl-*β*-sitosterols as an effective cytotoxic agent from the latex of *F. carica*, which has an inhibitory effect on the proliferation of various cancer cell lines in vitro [9]. Gond et al. reported that petroleum ether extract from the leaves of *F. carica* showed hepatoprotective properties [10]. Jeong et al. claimed that the methanol extract of *F. carica* had strong antibacterial activity against oral bacteria [11].

Tyrosinase catalyzes the rate-limiting step of melanogenesis, which hydroxylates tyrosine to 3,4-dihydroxyphenylalanine (DOPA) and converts DOPA through oxidation to dopaquinone [12,13]. Hyperpigmentation, postinflammatory pigmentation, melasma, and skin aging are all caused by excessive melanin pigment production and accumulation. As a result, tyrosinase inhibitors that reduce or inhibit melanin formation are gaining popularity in the pharmaceutical, cosmetics, and food industries [14]. Tyrosinase inhibitors are clinically useful for the treatment of certain skin diseases associated with melanin hyperpigmentation. Due to their effects on skin health, tyrosinase inhibitors are also used in the cosmetic industry [15–17].

It is known that the genome is exposed to many different factors that cause DNA damage. DNA damage causes many cellular events in the cell that can fight the damage or, if it is unable to do so, cause programmed cell death [18]. The cell responds to DNA damage with different metabolic pathways. DNA damages cause cell death by activating the apoptosis pathway or they can be corrected by repair mechanisms. The DNA molecule has a dynamic structure and is important because it is the only biomolecule that can be repaired [19,20].

In this paper, it was aimed to investigate the phytochemical characterization and biological properties (2,2-diphenyl-1-picrylhydrazyl (DPPH) radical scavenging activity, tyrosinase inhibitory, and DNA interaction properties) of *F. carica* leaf extracts in order to determine their potential for use in the treatment of various diseases. The chemical composition of the most active extract has been investigated to obtain information about the compounds that may be responsible for the activity in order to improve frontier knowledge in this area of research.

## 2. Materials and methods

### 2.1. Plant material

The leaf parts of *F. carica* L. were collected in August 2021 from Ortahisar, Geçit Village (height 600 m) of Trabzon province in the Eastern Black Sea Region of Turkey. Plant materials were identified by Assoc. Dr Gülin Renda.

### 2.2. Extraction

The leaves of *F. carica* used in the study were dried in a cool and moisture-free environment. The dried leaves were pulverized using a laboratory-type mechanical herb grinder to yield 100 g of plant material. Of the powdered plant material, 54.84 g was taken and transferred to a 1 L glass flask and 500 mL of 70% methanol solvent was added. It was extracted in a shaker for 4 h at 40 °C. The obtained extract was filtered through pleated filter paper. Four hundred milliliters of 70% methanol was added to the filtrated plant sample and extracted under the same conditions and filtered. The filtrates were combined and the solvent was evaporated to dryness under a rotary evaporator at 30–40 °C, and 11.08 g methanol extract (ME) (yield: 20.22%) was obtained. The crude methanol extract was then fractionated with *n*-hexane, dichloromethane, and *n*-butanol, respectively, to obtain extracts of different polarities. The following were obtained: 0.83 g *n*-hexane extract (HE) (yield: 7.48%), 0.61 g dichloromethane extract (DE) (yield: 5.50%), 0.44 g *n*-butanol extract (BE) (yield: 3.94%), and 1.67 g remaining water extract (WE) (yield: 15.10%).

### 2.3. Total phenolic content of extracts

The total phenolic content of the extracts was determined spectrophotometrically with the Folin-Coicalteu reagent. As a standard, different concentrations of gallic acid were used. Extracts were filled into the tubes. Subsequently, 0.5 N Folin-Ciocalteu reagent and sodium carbonate were added and the mixture was incubated for 30 min in the dark. At the end of this period, the absorbance of the mixtures was measured at 760 nm. The total phenolic contents of the samples are given as gallic acid equivalent (GAE)/dry weight of the extract [21].

### 2.4. LC–HRMS analysis

The liquid chromatography–high resolution mass spectrometry (LC–HRMS) experiments were performed on a Thermo Orbitrap Q-Exactive mass spectrometer (Bremen, Germany) in ESI (Electrospray ionization) Source equipped with a Troyasil C18 column (150 × 3 mm i.d., 3 mm particle size). One percent formic acid in water and 1% formic acid in methanol made up the mobile phases A and B, respectively. Zero to 3 min of 50% A and 50% B, 3.01–7 min of 100% B, and finally 7.01–15 min of 50% A and 50% B made up the gradient method. The mobile phase flow rate was 0.35 mL/min, and the column temperature was adjusted to 22 °C. Temperature and relative humidity were adjusted at 22.0 ± 5.0 °C and 50 ± 15% rh, accordingly [22]. The instrument’s high-resolution mode had a scanning ion range of m/z 85–1500. The MS parameters were used as follows: sheath gas flow rate: 45, aux gas flow rate 10, spray voltage 3.80, capillary temperature 320 °C, aux gas heater temperature 320 °C, and S-lens RF level 50. Comparing the retention times of standard compounds (with purity levels between 95% and 99%) with HRMS data from the Bezmiâlem Vakıf University, Drug Application and Research Center Library allowed for the identification of the compounds (ILMER). The details and validation parameters of the method and standards were given previously by Bektaş et al. [23].

The dried *n*-butanol extract was dissolved in the mobile phase (2.5 mL; A:B; 50:50; v/v), and then internal standard (100 mg/L; dihydrocapsaicin; 97%) was added to a final concentration of 3 ppm and volume was filled with mobile phase mixture up to 5 mL. The solution was then filtered through a 0.45-μm filter before being injected into the LC in a volume of 2 mL [24,25].

### 2.5. 2,2-diphenyl-1-picrylhydrazyl (DPPH) radical scavenging assay

The DPPH radical scavenging activities of the extracts were carried out using the spectrophotometric method [26]. First of all, the stock solution of the extracts dissolved in methanol was prepared as 10 mg/mL. Afterward, the extracts (25–200 μg/mL) were added to the DPPH solution (0.2 mM) (Sigma-Aldrich, D9132) in methanol and incubated for 30 min at room temperature in the dark. After incubation, absorbance at 517 nm was measured. The DPPH radical scavenging activities against increasing concentrations of the extracts were calculated from formula 1. Gallic acid (Sigma-Aldrich, G7384) was used as a positive control. Each experiment was performed six times.

Formula 1: % Inhibition = (A_control_ - A_extracts_) / A_control_ × 100 A_control_: Absorbance of DPPH solution; A_extracts_: Absorbance of DPPH solution after addition of extracts.

### 2.6. Tyrosinase inhibition assay

The tyrosinase enzyme inhibition efficiency of the extracts was determined using the spectrophotometric method [27]. Twenty microliters of each of the extracts (20–200 μg/mL) and 100 μL of phosphate buffer (pH 6.8) were added to the wells. Thereupon, 20 μL tyrosinase (250 U/mL) (Sigma, T3824) was added and incubated for 10 min. After this time, 20 μL of L-DOPA (3 mM) (Sigma-Aldrich, Sigma, D9628) was added and allowed to incubate for 10 min. After incubation, the absorbances were measured at 475 nm. Tyrosinase inhibition against increasing concentrations of extracts was calculated from formula 2. Kojic acid (Sigma-Aldrich, K3215) was used as a positive control in the study. Each experiment was performed six times. Formula 2: % Inhibition = (A_control_ - A_extracts_) / A_control_ × 100 A_control_: Absorbance of absence of the extracts; A_extracts_: Absorbance of presence of the extracts.

### 2.7. Supercoiled pBR322 plasmid DNA damage effects of extracts

Supercoiled pBR322 plasmid DNA damage effects of the extracts were performed using agarose gel electrophoresis. First of all, the stock solution of the samples dissolved in water was prepared as 10 mg/mL. In this study, the agarose gel well contents were formed to be 10 μL. pBR322 plasmid DNA (Thermo-Scientific, SD0041), buffer solution (50 mM Tris-HCl (pH 7)), and extracts (80–200 μg/mL) were added to an Eppendorf tube and incubated at 37 °C for 60 min. At the end of this period, this mixture was loaded into the agarose gel with loading dye. After the addition of the running buffer Tris-acetic acid-EDTA (TAE), the gel was run for 90 min (100 V, 400 mA). Gallic acid was used as a positive control. The obtained results were photographed with the BioRad Gel Doc XR system and were calculated with Image Lab Version 4.0.1 program [23,28].

### 2.8. Supercoiled pBR322 plasmid DNA damage protective effects of extracts

The supercoiled pBR322 plasmid DNA damage protective effects of the extracts were investigated using the agarose gel electrophoresis method.

Fenton’s reagent: In this study, 1 mM FeSO_4_ (Sigma-Aldrich, V000119) and 2% H_2_O_2_ (Sigma-Aldrich, 216763) were used to realize the Fenton reaction. The protective effects of the extracts against DNA damage caused by the hydroxyl radical were determined by supercoiled pBR322 plasmid DNA. Supercoiled pBR322 plasmid DNA, buffer solution (50 mM Tris-HCl (pH 7)), 1 mM FeSO_4_, 2% H_2_O_2_, and extracts (80–200 μg/mL) were added and incubated at 37 °C for 30 min and loaded into an agarose gel. The above electrophoresis procedures were carried out [23,28].

UV radiation: The protective effects of the extracts against DNA damage caused by UV radiation were determined using the method developed by Hahn et al. [29]. Supercoiled pBR322 plasmid DNA, buffer solution (50 mM Tris-HCl (pH 7)), and extracts (80–200 μg/mL) were added and exposed to UV radiation (254 nm) for 30 min. Subsequently, the mixtures were incubated at 37 °C for 30 min. The above electrophoresis procedures were carried out [28].

Methylene blue in the presence of light: Methylene blue is a dye that creates singlet oxygen and causes DNA damage when exposed to light [28,30]. Therefore, in this study, the potential to prevent DNA damage induced by methylene blue in the presence of light was investigated. Supercoiled pBR322 plasmid DNA, buffer solution (50 mM Tris-HCl (pH 7)), methylene blue (25 μM), and extracts (80–200 μg/mL) were added and exposed to white light for 45 min. Next, the mixtures were incubated at 37 °C for 30 min. The above electrophoresis procedures were carried out [28].

## 3. Results

### 3.1. Total phenolic contents

The determination of the total phenolic contents of the extracts was examined spectrophotometrically using the Folin-Coicalteu reagent. The results were calculated after drawing the standard gallic acid graph (R^2^ = 0.9971) and are given in Table 1. According to Table 1, the BE had the highest total phenolic content with 72.58 ± 4.52 mg GAE/g dry weight followed by WE (36.88 ± 5.64 mg GAE/g dry weight) and ME (33.72 ± 1.55 mg GAE/g dry weight).

### 3.2. LC–HRMS analysis of the n-butanol extract

As a result of the spectrophotometric analysis, LC–HRMS analysis of BE, which has the highest total phenolic content, was performed. It revealed the presence of 27 compounds. Retention time (Rt), found molecular ion, and quantity of each compound are presented in Table 2. The total amount of individual phenolic compounds found in BE was 50.62 g/kg. Rutin (40.13 g/kg) was the most abundant chemical, followed by fumaric acid (4.658 g/kg), hyperoside (1.767 g/kg), and chlorogenic acid (1.517 g/kg).

### 3.3. DPPH radical scavenging actions of the extracts

The DPPH radical scavenging activities of the extracts were carried out using the spectrophotometric method. The results are presented in Table 3. In this study, the radical scavenging efficiency of the extracts in the range of 20–200 μg/mL was investigated. As seen in Table 3, ME, HE, and BE showed radical scavenging activity depending on the increasing concentrations. BE showed 37.01 ± 1.15% and 82.57 ± 0.88% radical scavenging activity at 80 and 200 μg/mL, respectively, and it had the highest activity among extracts. Gallic acid, which was used as a positive control, showed higher radical scavenging efficiency than all extracts.

### 3.4. Tyrosinase inhibitory effects of the extracts

The extracts’ tyrosinase inhibitory effects were measured using a spectrophotometric method. Table 4 displays the results. As shown in Table 4, BE had the highest inhibitory effect, with inhibitory percentages of 17.06 ± 0.34% and 66.00 ± 0.04% at 25 and 200 μg/mL, respectively, whereas ME had 58.50 ± 2.86% inhibition at 200 μg/mL.

### 3.5. Supercoiled pBR322 plasmid DNA damage properties of extracts

**Figure 1:**
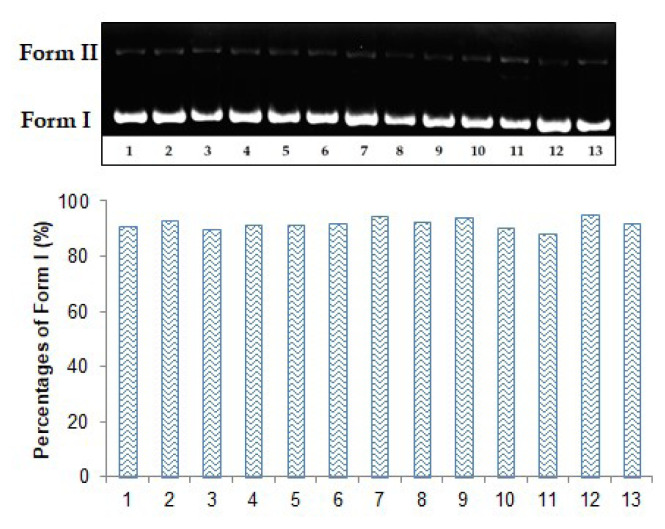
The hydrolytic nuclease activities of the extracts on plasmid pBR322 DNA. Lane 1: DNA control; lane 2: DNA + 80 μg/mL ME; lane 3: DNA + 200 μg/mL ME; lane 4: DNA + 80 μg/mL HE; lane 5: DNA + 200 μg/mL HE; lane 6: DNA + 80 μg/mL DE; lane 7: DNA + 200 μg/mL DE; lane 8: DNA + 80 μg/mL BE; lane 9: DNA + 200 μg/mL BE; lane 10: DNA + 80 μg/mL WE; lane 11: DNA + 200 μg/mL WE; lane 12: DNA + 80 μg/mL gallic acid; lane 12: DNA + 200 μg/mL gallic acid.

Using agarose gel electrophoresis, the hydrolytic nuclease activity of samples against plasmid pBR322 DNA was determined. The BioRad Gel Doc XR system was used to visualize the results, and the percentages of band intensities were calculated using the Image Lab Version 4.0.1 program. The electrophoresis image was given in Figure 1. Gallic acid was used as a positive control in the study. As shown in Figure 1, the band intensities of Form I was found to be similar to each other. Form I percentage of DNA control was 90.90%. In addition to the extracts, Form I in all bands did not alter significantly and remained between 90% and 95%. In this situation, it was determined that extracts at 80 and 200 μg/mL did not cause DNA damage to plasmid pBR322.

### 3.6. Supercoiled pBR322 plasmid DNA damage protective effects of extracts

**Figure 2:**
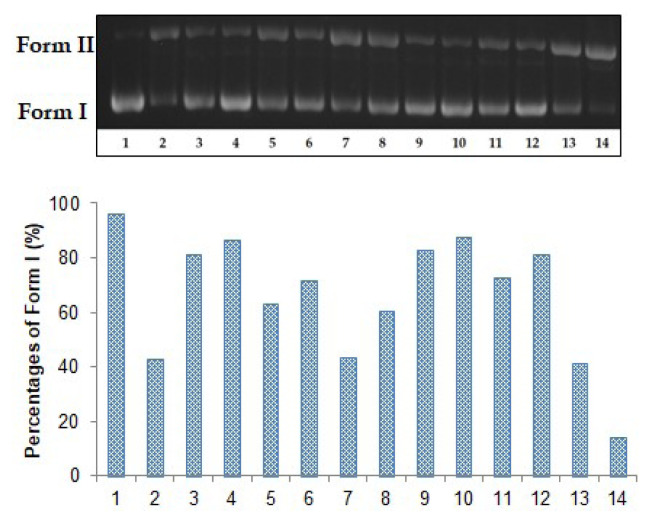
The plasmid pBR322 DNA protective effects of the extracts against Fenton’s reagents. Lane 1: DNA control; lane 2: DNA + 1 mM FeSO_4_ + %2 H_2_O_2_; lane 3: DNA + 80 μg/mL ME + 1 mM FeSO_4_ + %2 H_2_O_2_; lane 4: DNA + 200 μg/mL ME + 1 mM FeSO_4_ + %2 H_2_O_2_; lane 5: DNA + 80 μg/mL HE + 1 mM FeSO_4_ + %2 H_2_O_2_; lane 6: DNA + 200 μg/mL HE + 1 mM FeSO_4_ + %2 H_2_O_2_; lane 7: DNA + 80 μg/mL DE + 1 mM FeSO_4_ + %2 H_2_O_2_; lane 8: DNA + 200 μg/mL DE + 1 mM FeSO_4_ + %2 H_2_O_2_; lane 9: DNA + 80 μg/mL BE + 1 mM FeSO_4_ + %2 H_2_O_2_; lane 10: DNA + 200 μg/mL BE + 1 mM FeSO_4_ + %2 H_2_O_2_; lane 11: DNA + 80 μg/mL WE + 1 mM FeSO_4_ + %2 H_2_O_2_; lane 12: DNA + 200 μg/mL WE + 1 mM FeSO_4_ + %2 H_2_O_2_; lane 13: DNA + 80 μg/mL gallic acid + 1 mM FeSO_4_ + %2 H_2_O_2_; lane 14: DNA + 200 μg/mL gallic acid + 1 mM FeSO_4_ + %2 H_2_O_2_.

**Figure 3:**
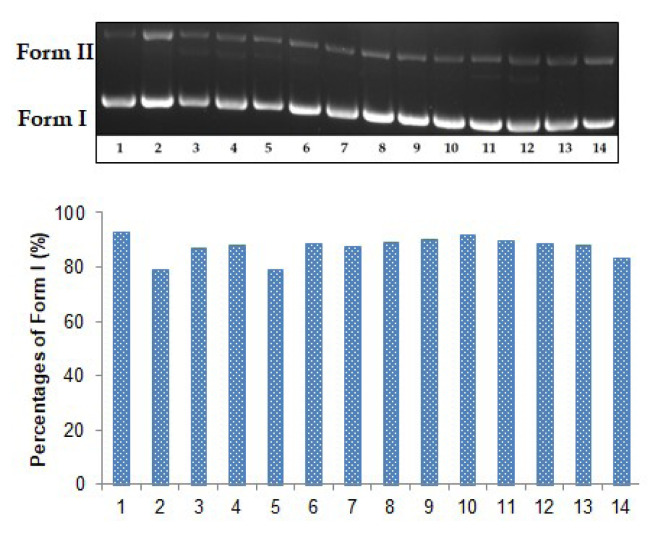
The plasmid pBR322 DNA protective effects of the extracts against UV radiation. Lane 1: DNA control; lane 2: DNA + UV radiation (366 nm, 30 min); lane 3: DNA + 80 μg/mL ME + UV radiation (366 nm, 30 min); lane 4: DNA + 200 μg/mL ME + UV radiation (366 nm, 30 min); lane 5: DNA + 80 μg/mL HE + UV radiation (366 nm, 30 min); lane 6: DNA + 200 μg/mL HE + UV radiation (366 nm, 30 min); lane 7: DNA + 80 μg/mL DE + UV radiation (366 nm, 30 min); lane 8: DNA + 200 μg/mL DE + UV radiation (366 nm, 30 min); lane 9: DNA + 80 μg/mL BE + UV radiation (366 nm, 30 min); lane 10: DNA + 200 μg/mL BE + UV radiation (366 nm, 30 min); lane 11: DNA + 80 μg/mL WE + UV radiation (366 nm, 30 min); lane 12: DNA + 200 μg/mL WE + UV radiation (366 nm, 30 min); lane 13: DNA + 80 μg/mL gallic acid + UV radiation (366 nm, 30 min); lane 14: DNA + 200 μg/mL gallic acid + UV radiation (366 nm, 30 min).

**Figure 4:**
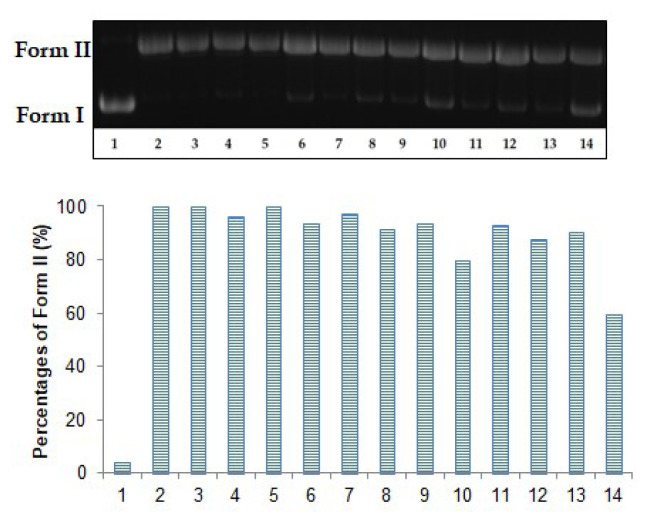
The plasmid pBR322 DNA protective effects of the extracts against MB with light irradiation. Lane 1: DNA control + white light (45 min); lane 2: DNA + 25 μM MB + white light (45 min); lane 3: DNA + 80 μg/mL ME + 25 μM MB + white light (45 min); lane 4: DNA + 200 μg/mL ME + 25 μM MB + white light (45 min); lane 5: DNA + 80 μg/mL HE + 25 μM MB + white light (45 min); lane 6: DNA + 200 μg/mL HE + 25 μM MB + white light (45 min): DNA + 80 μg/mL DE + 25 μM MB + white light (45 min); lane 8: DNA + 200 μg/mL DE + 25 μM MB + white light (45 min); lane 9: DNA + 80 μg/mL BE + 25 μM MB + white light (45 min); lane 10: DNA + 200 μg/mL BE + 25 μM MB + white light (45 min); lane 11: DNA + 80 μg/mL WE + 25 μM MB + white light (45 min); lane 12: DNA + 200 μg/mL WE + 25 μM MB + white light (45 min); lane 13: DNA + 80 μg/mL gallic acid + 25 μM MB + white light (45 min); lane 14: DNA + 200 μg/mL gallic acid + 25 μM MB + white light (45 min).

The supercoiled pBR322 plasmid DNA damage protective effects of the extracts against Fenton’s reagent, UV radiation, and singlet oxygen were carried out using the agarose gel electrophoresis method. It is known that the supercoiled form (Form I) migrates most rapidly when the supercoiled plasmid DNA is subjected to electrophoresis. After interaction with compounds/extracts, a nicked form (Form II) occurs with the break in a single chain and this form moves the lowest migration. If there is a break in the double chain, a linear form (Form III) occurs. The formed form migrates between Forms I and II [31]. Electrophoresis images are given in Figures 2–4. According to the results, Form I was determined as 96.10% (Figure 2, lane 1). Subsequently, Form I reduced to 42.70% when plasmid pBR322 DNA was exposed to Fenton’s reagent (Figure 2, lane 2). It was observed that Form I increased with the addition of the extracts. BE showed the highest protective effect among the extracts, whilst DE had the lowest efficiency. In the presence of BE, Form I was determined as 82.90% and 87.30%, respectively (Figure 2, lanes 9 and 10). In addition, Form I was calculated as 81.10% and 86.60% in the presence of ME (Figure 2, lanes 3 and 4).

In UV irradiation protective studies, Form I was 93.00% (Figure 4, lane 1). Subsequently, Form I decreased to 79.10% when plasmid pBR322 DNA was exposed to UV light (Figure 3, lane 2). Form I was determined as 90.40% and 91.60%, respectively (Figure 3, lanes 9 and 10), in the presence of BE which had the highest protective effects against UV radiation.

In singlet oxygen protective studies, Form I was found to be 96.20% (Figure 4, lane 1). Form I disappeared completely when plasmid pBR322 DNA was stimulated with methylene blue in the presence of light (Figure 4, lane 2). The results showed that Form I was enhanced in small amounts with the extracts. The BE displayed the highest protective effect among the extracts in this study. In the presence of BE, the density of Form I was determined as 6.60% and 20.70%, respectively (Figure 4, lanes 9 and 10).

## 4. Discussion

*F. carica*, popularly known as fig, was one of the earliest plants that humans cultivated. It is an important product that is consumed both dry and fresh all around the world. Various parts of fig have been used as antiinflammatory and antispasmodic in traditional medicine, as well as for the treatment of gastrointestinal, respiratory, and cardiovascular disorders [3]. Phytochemical studies on *F. carica* reveal that many compounds have been isolated from different parts of it. In addition, its various parts have been shown to have numerous pharmacological and biological activities but the number of studies on the leaves is limited [4]. Despite all these, the DNA interaction study on the plant has not been found in the current literature. The purpose of this research was to search for the connection between the biological activities of *F. carica* and its components, as well as providing basic evidence for herbal preparations that can be obtained from the plant. In addition, the results of the activities of the extracts obtained by the method used in this study were compared with the values in the literature.

In this study, BE showed the highest total phenolic content with 72.58 ± 4.52 mg GAE/g dry weight, and the total phenolic content of ME was found as 33.72 ± 1.55 mg GAE/g dry weight. Ghazi et al. reported that the total phenolic content of methanol extract of *F. carica* leaves from Saudi Arabia was 412.37 ± 57.90 mg GAE/100 g dry weight [32]. In another study, Ergül et al. reported that methanol extract of *F. carica* leaves from Saklıkent/Fethiye was determined as 16.11 mg GAE/g dry weight [33]. This result can be explained by the difference in extraction methods used. The effect of plant material collection time on the results is also noteworthy as another research topic.

The DPPH radical scavenging method is one of the fast and inexpensive methods to measure antioxidant activity [34]. The reduction of the DPPH radical with a compound or an extract that has a tendency to donate hydrogen atoms is the basis for this method. A spectrophotometer is used to measure absorbance at 517 nm, and any decrease in absorbance is interpreted as antioxidant activity. When the DPPH radical scavenging activities of the extracts were examined in this study, it was discovered that ME showed 47.60 ± 1.86% DPPH radical scavenging activity at the dose of 200 μg/mL. According to Ali et al., *F. carica* methanol extract was 40.30 ± 0.82%, 56.10 ± 1.45%, and 71.20 ± 1.68% at 150, 200 and 250 μg/mL, respectively in DPPH assay [35]. In another study, Ayoub et al. reported that the radical scavenging effects of the methanol extract of *F. carica* leaves were found to be between 11.31 ± 3.86% and 87.03 ± 0.15% at 50–1000 μg/mL [36]. Comparing the studies in the literature with our study, it was revealed that *F. carica* extracts showed similar DPPH radical scavenging activity. In addition, total phenolic content and DPPH radical scavenging activity were compatible in terms of effect. The results also showed that BE was the extract with both the highest total phenolic content and DPPH radical scavenging efficiency among the subextracts obtained from the main methanol extract.

Tyrosinase is found in fungi, animals, and plants; and it catalyzes the hydroxylation of monophenols to *o*-diphenols and the oxidation of *o*-diphenols to *o*-quinones using molecular oxygen [37]. In this study, tyrosinase inhibitory properties of the extracts were investigated by the spectrophotometric method. Among extracts, BE had the highest inhibitory effect in the range of 17.06 ± 0.34% and 66.00 ± 0.04% at 25 and 200 μg/mL against tyrosinase and methanol extract also showed 58.50 ± 2.86% tyrosinase inhibitory effects at 200 μg/mL. Rafiq et al. reported antityrosinase activity of the methanol extract of the *F. carica* and IC_50_ values of leaves and fruit extracts were 156.20 ± 12 μg/mL and 132.00 ± 10.5 μg/mL, respectively [38]. Meziant et al. reported that IC_50_ values of *F. carica* extracts ranged from 95.08 and 447.49 μg/mL [8]. When compared to previous studies, our extracts showed similar tyrosinase inhibitory effects due to phenolic contents. The fact that the activity of BE was higher than that of ME indicates that substances not responsible for the effect were removed during fractionation and that the components responsible for the tyrosinase inhibitory effect were collected in BE at a higher rate.

DNA in normal cells is constantly exposed to DNA-damaging agents from endogenous sources (reactive oxygen species, free radicals from normal metabolism, and internal replication mistakes) or exogenous sources (UV radiation, exposure to chemicals) [39]. Failure to repair the damages in DNA can lead to genomic instability and as a result, many diseases such as premature aging, immunodeficiency, neurological anomalies, and cancer may occur [40,41]. In this study, it is aimed to protect against the DNA damage caused by various sources and we investigated using supercoiled pBR322 plasmid DNA by agarose gel electrophoresis.

The reduction of Fe^3+^ with superoxide to be Fe^2+^ and the reaction of Fe^2+^ with H_2_O_2_ to form the hydroxyl radical is known as the Fenton reaction. The hydroxyl radical is known to be the most damaging species among reactive oxygen species due to its strong reaction with biomolecules [42]. UV radiation disrupts the structure of DNA, causes bending, and thus inhibits transcription and replication [43]. Methylene blue is an effective photosensitizer compound. There are studies in the literature that methylene blue produces singlet oxygen and causes damage to DNA in the presence of light [28,30]. The BE showed the highest protective effect among the extracts owing to its phenolic contents. To the best of our knowledge, there was not any report against supercoiled pBR322 plasmid DNA damage protective effects of *F. carica*. However, Lightbourn and Thomas reported that fig leaf aqueous extract inhibited diethylstilbestrol-induced DNA singlestrand breakage in human breast epithelial cells using comet assay, but content analysis was not performed in their study [44]. In our present work, studies were carried out using methanol, hexane, dichloromethane, butanol, and water extracts.

According to LC–HRMS analysis, 27 phenolic compounds were identified and the most abundant compound was rutin (40.13 g/kg) followed by fumaric acid (4.658 g/kg), hyperoside (1.767 g/kg), and chlorogenic acid (1.517 g/kg). In a study from Turkey, α-tocopherol content of the *n*-hexane extract was found to be 0.057% on the dry-weight basis of *F. carica* leaves [45]. Methanol and water extracts of *F. carica* leaf extracts from Turkey were investigated using GC-MS and the most abundant components of the methanol extract were found as benzene, 4-methyl-1,4-heptadiene (6.85%), 1-pentene, 2,3-dimethyl-(2.72%) for the water extract, they were 2*H*-furo[2,3-*H*]-1-benzopyran-2-one (53.64%), bergapten (19.27%), 9,12,15-octadecatrienoic acid, methyl ester, (Z,Z,Z)-(4.05%) [33]. In another study conducted with the same method, the main constituents of the fig leaves extract were ethanol (94.36%), 6-methylthiol-benzothienoquinoline (0.94%), and methyl benzoylformate (0.84%) [46]. Since fractionation was not performed in these studies and the methods used did not allow the identification of phenolic substances, there is no similarity with the results obtained in our study. As a result of LC-MS analysis of leaf extracts of 18 cultivars of *F. carica* collected from Malaysia, the ‘Violette solise’ cultivar was found to have the highest antioxidant activity and contain the highest phenolic contents and the presence of phenolic acids (dihydroxybenzoic acid di-pentoside, caffeic acid, caffeoylmalic acid, coumaroylmalic acid, ferulic acid malate, and psoralic acid glucoside), C-glycosides of flavones, rutin and prenylgenistein in this cultivar was reported [47]. HPLC/DAD and HPLC/UV were used to profile the metabolites in the leaves, pulps, and peels of two Portuguese white varieties of *F. carica*. The phenolic profile of leaf samples was composed of 3-*O*- and 5-*O*-caffeoylquinic acids, ferulic acid, isoquercetin, rutin, psoralen, and bergapten, and organic acids (oxalic, citric, malic, quinic, shikimic, and fumaric acid) [4]. The results of our study support the literature in terms of the presence of rutin, but it is remarkable that hyperoside, one of the major compounds, was not found in the samples examined in these studies.

The tyrosinase inhibitory and DNA damage protective effects of BE might be due to the presence of major compounds namely rutin, fumaric acid, hyperoside, and chlorogenic acid. Rutin is used in human nutrition and medicine due to its numerous pharmacological properties. It was reported to inhibit tyrosinase significantly, with an IC_50_ value of the enzyme of 0.13 ± 0.003 mM. Rutin has been reported to be a potent antipigment agent due to the presence of hydroxyl groups [48]. Fumaric acid was shown to be a reversible inhibitor of tyrosinase with a parabolic noncompetitive inhibition mechanism with IC_50_ =13.7 ± 0.25 mM and K_islope_ =12.64 ± 0.75 mM [49]. It has been shown that hyperoside, which has a flavonoid structure, has a stronger tyrosinase inhibitory effect than kojic acid (80 ± 17 μg/mL), and its IC_50_ value is below 1 μg/mL [50]. Haydar and Çelik reported that rutin protects the pBR322 plasmid DNA from idarubicin-induced DNA strand breaks at 2 mM (92.50%) [51]. Xu et al. investigated DNA protective activities of chlorogenic acid isomers including 3-*O*-caffeoylquinic acid, 4-*O*caffeoylquinic acid, and 5-*O*-caffeoylquinic acid, and the results showed that the compounds exhibited DNA damage protective effect induced by Fenton’s reagents [52]. Liu reported that hyperoside protected the cells against damage induced by H_2_O_2_ by reducing reactive oxygen species [53].

## 5. Conclusion

In this work, BE showed the highest total phenolic content, DPPH radical scavenging activity, and tyrosinase inhibition. Our study showed that both the extraction method used and the subsequent fractionation step were suitable for obtaining an extract with high tyrosinase inhibitory activity. The extracts did not damage supercoiled pBR322 plasmid DNA, but they protected against DNA damage caused by hydroxyl radical, UV radiation, and singlet oxygen, especially BE. In addition, 27 phenolic compounds were identified in the presence of BE and rutin was the most abundant compound. Studies on rutin, which is the substance with the highest amount in the most active extract, show that it has an important role in the activity. Antioxidants are effective compounds in health protection since they are frequently mentioned as compounds that reduce the risk of chronic diseases, such as cancer and cardiac cancer diseases, which have increased in recent years. These findings revealed the potential of using BE, which is rich in antioxidants, in the treatment of various diseases and indicated that additional research can be planned on products that can be prepared from BE.

## Figures and Tables

**Table 1:** Total phenolic contents of the extracts (mg GAE/g dry weight).

	mg GAE/g dry weight
**Methanol extract (ME)**	33.72 ± 1.55
***n*** **-Hexane extract (HE)**	29.93 ± 1.27
**Dichloromethane extract (DE)**	28.38 ± 2.19
***n*** **-Butanol extract (BE)**	72.58 ± 4.52
**Water extract (WE)**	36.88 ± 5.64

**Table 2:** Quantitative determination (g/kg) of phytochemicals in *n*-butanol extract.

Compounds	Rt (min)	Found *m/z* (molecular ion)	Quantity g/kg	U %*
Ascorbic acid	2.24	175.0239	0.044	3.94
Chlorogenic acid	2.53	353.0860	1.517	3.58
Fumaric acid	2.20	115.0031	4.658	2.88
Chicoric acid	3.00	473.0702	0.049	2.28
Orientin	3.03	447.0910	0.852	3.67
Caffeic acid	3.02	179.0341	0.122	3.74
Luteolin-7-rutinoside	3.75	593.1482	0.014	3.06
Vanillic acid	3.91	167.0341	0.588	3.49
Naringin	3.91	579.1690	0.006	4.20
Rutin	4.20	609.1431	40.13	3.07
Rosmarinic acid	4.56	359.0754	0.003	3.77
Hyperoside	4.37	463.0859	1.767	3.46
Dihydrokaempferol	4.72	287.0547	0.001	2.86
Oleuropein	4.80	539.1743	0.061	4.08
Quercitrin	5.07	447.0910	0.128	3.78
Scutellarein	5.76	285.0390	0.052	2.84
Quercetin	5.63	301.0339	0.008	2.95
Herniarin	5.41	177.0537	0.039	3.89
Salicylic acid	5.67	137.0237	0.258	1.89
Naringenin	5.68	271.0598	0.003	4.20
Luteolin	5.76	285.0390	0.041	3.42
Genistein	5.82	269.0442	0.007	3.28
Apigenin	6.07	269.0442	0.005	2.87
Hispidulin	6.04	301.0692	0.045	3.41
Gypsogenic acid	7.74	485.3248	0.034	3.34
Homogentisic acid	2.47	167.0341	0.155	4.35
Dihydrocaffeic acid	2.84	181.0497	0.024	0.86

* Uncertainty values (k = 2, 95% confidence interval)

**Table 3:** DPPH radical scavenging activities of the extracts (%).

	20 μg/mL	40 μg/mL	80 μg/mL	200 μg/mL
**ME**	4.22 ± 1.83	13.91 ± 2.66	21.37 ± 2.65	47.60 ± 1.86
**HE**	4.88 ± 0.46	10.01 ± 0.39	17.31 ± 0.64	42.62 ± 1.66
**DE**	-	4.40 ± 1.58	12.92 ± 1.49	29.22 ± 2.43
**BE**	8.81 ± 1.79	17.98 ± 0.40	37.01 ± 1.15	82.57 ± 0.88
**WE**	-	6.52 ± 0.25	12.54 ± 1.78	35.67 ± 3.00
**Gallic acid**	92.63 ± 0.11	92.55 ± 0.19	92.79 ± 0.15	92.78 ± 0.14

**Table 4:** Tyrosinase inhibitory effects of the extracts (%).

	25 μg/mL	50 μg/mL	100 μg/mL	200 μg/mL
**ME**	19.67 ± 1.05	24.04 ± 3.23	43.92 ± 3.22	58.50 ± 2.86
**HE**	14.56 ± 1.09	21.76 ± 0.53	37.55 ± 9.35	52.32 ± 5.69
**DE**	-	18.50 ± 2.30	24.63 ± 5.81	45.60 ± 5.51
**BE**	17.06 ± 0.34	37.22 ± 4.48	63.91 ± 2.80	66.00 ± 0.04
**WE**	-	-	19.89 ± 1.64	46.33 ± 2.05
**Kojic acid**	90.82 ± 0.21	97.20 ± 0.47	98.44 ± 0.98	98.97 ± 0.22
